# Evaluation of Arabian Vascular Plant Barcodes (rbcL and matK): Precision of Unsupervised and Supervised Learning Methods towards Accurate Identification

**DOI:** 10.3390/plants10122741

**Published:** 2021-12-13

**Authors:** Rahul Jamdade, Maulik Upadhyay, Khawla Al Shaer, Eman Al Harthi, Mariam Al Sallani, Mariam Al Jasmi, Asma Al Ketbi

**Affiliations:** 1 Sharjah Seed Bank and Herbarium, Environment and Protected Areas Authority, Sharjah P.O. Box 2926, United Arab Emirates; khawla.alali@epaa.shj.ae (K.A.S.); eman.khalid@epaa.shj.ae (E.A.H.); mariam.alsallani@epaa.shj.ae (M.A.S.); mariam.aljasmi@epaa.shj.ae (M.A.J.); asma.alhafri@epaa.shj.ae (A.A.K.); 2 Population Genomics Group, Department of Veterinary Sciences, Ludwig Maximillians University, 80539 Munich, Germany; U.Maulik@gen.vetmed.uni-muenchen.de

**Keywords:** Arabian Peninsula, plant DNA barcoding, unsupervised method, supervised learning, alignment and alignment-free analysis

## Abstract

Arabia is the largest peninsula in the world, with >3000 species of vascular plants. Not much effort has been made to generate a multi-locus marker barcode library to identify and discriminate the recorded plant species. This study aimed to determine the reliability of the available Arabian plant barcodes (>1500; rbcL and matK) at the public repository (NCBI GenBank) using the unsupervised and supervised methods. Comparative analysis was carried out with the standard dataset (FINBOL) to assess the methods and markers’ reliability. Our analysis suggests that from the unsupervised method, TaxonDNA’s All Species Barcode criterion (ASB) exhibits the highest accuracy for rbcL barcodes, followed by the matK barcodes using the aligned dataset (FINBOL). However, for the Arabian plant barcode dataset (GBMA), the supervised method performed better than the unsupervised method, where the Random Forest and K-Nearest Neighbor (gappy kernel) classifiers were robust enough. These classifiers successfully recognized true species from both barcode markers belonging to the aligned and alignment-free datasets, respectively. The multi-class classifier showed high species resolution following the two classifiers, though its performance declined when employed to recognize true species. Similar results were observed for the FINBOL dataset through the supervised learning approach; overall, matK marker showed higher accuracy than rbcL. However, the lower rate of species identification in matK in GBMA data could be due to the higher evolutionary rate or gaps and missing data, as observed for the ASB criterion in the FINBOL dataset. Further, a lower number of sequences and singletons could also affect the rate of species resolution, as observed in the GBMA dataset. The GBMA dataset lacks sufficient species membership. We would encourage the taxonomists from the Arabian Peninsula to join our campaign on the Arabian Barcode of Life at the Barcode of Life Data (BOLD) systems. Our efforts together could help improve the rate of species identification for the Arabian Vascular plants.

## 1. Introduction

The Arabian Peninsula is the largest peninsula in the world and consists of nine countries. Saudi Arabia is the largest country (830,000 m^2^) that covers almost four-fifths of the Arabian Peninsula [1], whereas Bahrain is the smallest country (295.5 m^2^). In the case of plant species diversity estimates, there are more than 3500 native plants in the Arabian Peninsula [2]. Accordingly, Iraq exhibits the most diverse flora with more than 3300 species [3], followed by Yemen (number of species (*n*) = 2838) [4], Jordan (*n* = +2500) [5], Saudi Arabia (*n* = 2282) [6], Oman (*n* = 1239) [7], UAE (*n* = 731) [8], Kuwait (*n* = 407) [9], Qatar (*n* = 400) [10] and Bahrain (*n* = 307) [11].

**Figure 1:**
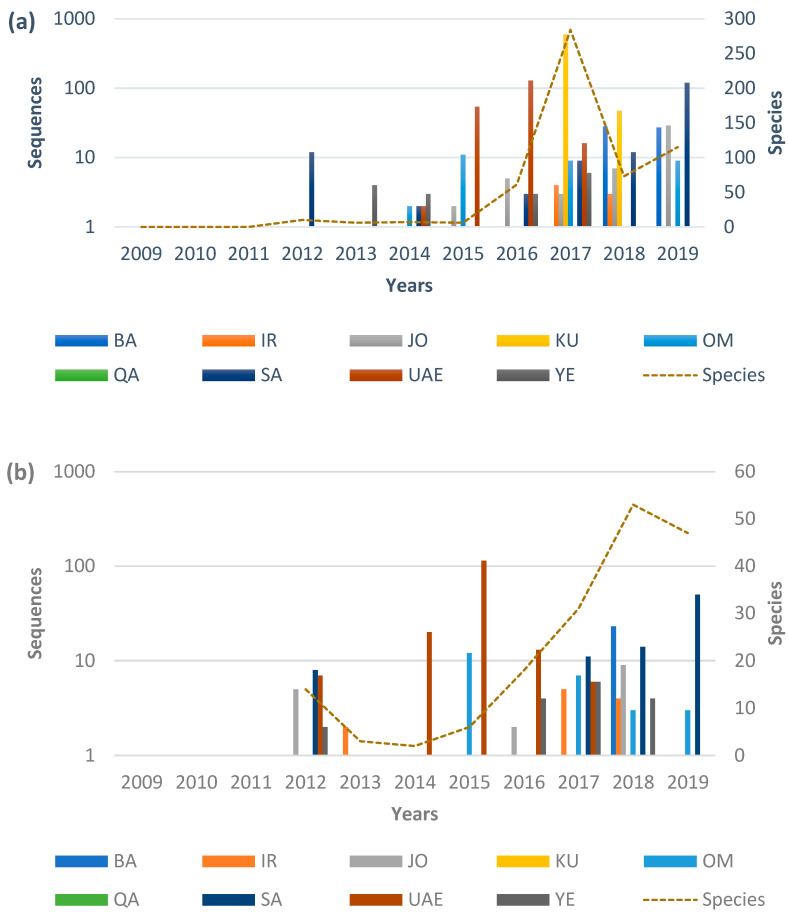
Sequences submitted over a decade at NCBI GenBank from the Arabian Peninsula. (**a**) rbcL and (**b**) matK. (Abbreviations: SA: Saudi Arabia, JO: Jordan, IR: Iraq, KU: Kuwait, BA: Bahrain, QA: Qatar, UAE: United Arab Emirates, OM: Oman, YE: Yemen).

Along with the plant species diversity studies, efforts towards generating multi-locus marker sequences called DNA barcodes have been undertaken in the last 10 to 12 years to assist in the identifying and discriminating those plant species. DNA barcoding is one of the most powerful tools that aims to use the information from single or multi-locus genes to identify species from higher (familial) to the lower (species) taxonomic levels, which could be otherwise difficult to identify using traditional morphological characters [12,13]. Since the DNA barcoding technique has been implemented in Arabia for over a decade, the highly sequenced loci was rbcL followed by matK [14,15]. The highest number of rbcL sequences were deposited by Kuwait (*n* = 592) in 2017 (Figure 1a), belonging to 243 species. Also, the highest number of species (*n* = 284) were deposited in this year (2017) (Figure 1a), where the major contribution was made by Kuwait (*n =* 243). In the case of matK, the UAE was the highest contributing country in 2015, depositing about 162 sequences (Figure 1). Considering the highest number of species sequenced, about six Arabian countries contributed matK sequences belong to 53 species in 2018 (Figure 1b). Moreover, in the following year (2019), the Saudi Arabia alone deposited about 50 sequences belonging to 42 species. Overall, rbcL remained to be the highly sequenced locus in Arabia followed by matK. The rbcL and matK loci are considered the most efficient molecular markers for delineating plant species; hence they have been nominated as the core DNA barcode marker by the Consortium for the Barcode of Life (CBOL) plant working group [16,17,18].

However, species discrimination has been more challenging in plants, as the success rate for these core barcode markers is not more than 70% (Consortium for the Barcode of Life (CBOL) plant working group) [16]. Significant studies have proven that no single barcode marker is capable of providing 100% species discrimination; hence, multi-locus combinations are usually required for achieving considerable species resolution [19,20,21,22]. There are several studies from Peninsular Arabia reporting the use of core barcode markers (rbcL and matK) (Figure 1), along with multi-locus markers for the identification and discrimination of plant taxa. For example, Al Qurainy et al. [23,24,25,26,27] used supportive markers (ITS, rpoB, rpoC1, PSBA-trnH, and rps16) as well as the core DNA barcode markers (rbcL and matK) on SA plant resources. Bafeel et al. [23,24,25] efficiently used rbcL and matK markers for studying arid plants. Khan et al. [26,27] used ITS, rpoB, rpoC1, psbA-trnH, and rps-16, along with the core barcode markers. From the most contributing country Kuwait, rbcL and ITS2 were reported to have better performance in assigning the specimens to their families and genera [9]. Studies from the UAE exhibit efficient use of rpoC1, psbK-psbI, rbcL and matK barcode markers for discrimination of the arid plants [28,29,30,31].

Along with selecting efficient DNA barcode markers for species identification, it is essential to select the most reliable method for DNA barcode sequence analysis. Various methods can classify species based on DNA sequence similarity. These are unsupervised clustering or OTU (Operational Taxonomic Unit) picking methods and supervised machine learning classification methods. The most commonly used method is the Barcode Gap analysis which refers to the separation between mean intra- and inter-specific sequence variability [32]. This form of analysis has been regularly mentioned in articles promoting barcoding to a broader audience [33,34,35]. However, there have been many arguments on exaggerating the barcode gap, further misleading the taxonomic classification [32,36,37,38,39]. Another tool is a web application, the Automated Barcode Gap Analysis (ABGD) [40], which uses a range of prior intraspecific divergence from data as a limit, then detects the barcode gap as the first significant gap beyond this limit and uses it to partition the data. There are also several similar standalone applications; one of them is TaxonDNA. It analyzes DNA sequences using the intra- and interspecific pairwise genetic distances to provide Best Match (BM), Best Close Match (BCM), and All Species Barcodes (ASB) [37]. These are the most common software tools for species delineation and are employed for the DNA barcode datasets.

However, besides these conventional methods, the supervised machine learning classifiers could provide high species resolution to improve initial confidence in DNA barcoding results [41]. Usually, the conventional methods need DNA sequence alignment or coding regions of the DNA. The variable (unalignable) or non-coding regions are treated as missing data or given other codes leading to an additional assumption [42]. On the other hand, machine learning methods could be employed for the aligned and unaligned dataset through data preprocessing, where character strings can be converted into numeric vector form or by generating numerical frequencies (K-mers) [41,43]. After that, the dataset could be used to test the accuracy of the machine learning classifiers through supervised classification, where the reference or training dataset is analyzed against the tested set, either by providing the reference dataset or by dedicating it for cross-validation. Cross-Validation (CV) is an empirical technique used to assess the generalizability of a classification method [44,45]. In this technique, the data is partitioned into mutually exclusive sub-sets or folds of approximately equal size, and each sub-set is tested against the other to estimate classification [46,47]. The CV approach exhibits a higher average performance than any single classification strategy [44]. However, it could be no more or less a form of bias if applied inappropriately [44]. As previously demonstrated, the supervised learning methods are promising candidates for species discrimination that provide high resolution to obtain excellent classification performances [41].

This study aimed to determine the reliability of the available Arabian plant barcodes at the public repository (NCBI GenBank). This could reveal the current status of Arabian plant barcodes, and aid future taxonomists in making the efforts required in creating the robust barcode library for the Arabian plants. This study assessed all available plant barcodes (rbcL and matK) deposited from Arabian countries. These barcodes were analyzed for their accuracy to assign the species to their concerned taxa using ad-hoc DNA barcode analysis methods, such as OTU picking and Supervised Machine Learning (SML). The OTU picking methods were employed using the TaxonDNA and ABGD, while SML methods were employed using an alignment and alignment-free approach (Logical Alignment-Free algorithm (LAF) [43], gappy kernel (GK) [42] and mismatch kernel (MK) [48]).

Further, various classifiers were employed (K-Nearest Neighbor (K-NN) [49], Random Forest (RF) [50], Support Vector Machine’s Sequential minimal optimization (SMO) classifier [51,52], and Multi-Class Classifier (MCC)) [53], for classification through a well-defined workflow. Thus, we demonstrated the robustness of all methods and markers employed with the standardized dataset with an almost similar number of sequences and species. Therefore, the observational data was analyzed with the standardized dataset (DS-FBPL1) obtained from the Barcode of Life Data (BOLD) Systems and we proposed a workflow that could better understand the efficiency of those methods and barcode markers employed for species identification.

## 2. Results

### 2.1. Data

We assessed plant DNA Barcode markers rbcL and matK from empirical data at NCBI GenBank from the Arabian Peninsula. Overall, 1502 DNA sequences with >400 bp were obtained and subjected to curation. The curated dataset was labelled as GBMA; it consisted of 1118 sequences belonging to the rbcL marker, representing 414 species, and 277 sequences belonging to the matK marker, representing 113 species [https://dx.doi.org/10.6084/m9.figshare.12190965, accessed on 22 September 2021]. Besides the GBMA dataset, the standardized dataset (FINBOL) was prepared from the DS-FBPL dataset available at the BOLD Systems to test the robustness of the methods and markers employed. The FINBOL dataset was further subjected to curation to finally achieve a dataset with enough species memberships (≥3 individuals per species) that are common in both the barcode markers (rbcL and matK) respectively. Overall, the FINBOL dataset consisted of 1194 sequences belonging to 382 species for each rbcL and matK barcode marker.

The rbcL barcode marker of the Arabian plant barcode dataset (GBMA) possesses most of the sequences belonging to class Magnoliopsida (75.13%), followed by Liliopsida (24.23%), where Poaceae is the dominating family with the highest number (*n*) of genera (*n* = 45) followed by Fabaceae (*n* = 40) and Asteraceae (*n* = 39). The Convolvulus was the highest contributing genera with 23 species. Similarly, the matK dataset contains most sequences belonging to class Magnoliopsida (49.81%) and Liliopsida (49.81%), where the Convolvulaceae was the most contributing family with 20 species. The date palm, *‘Phoenix dactylifera’* had the highest number of conspecifics, contributing 11.35% and 48.37% to the rbcL and matK datasets, respectively. The sequences belonging to rbcL and matK were of uneven length, while matK possessed indels with few insertions and deletions without stop codons.

### 2.2. Unsupervised Species Identification and Barcode Validation

The GBMA dataset was subjected to species discrimination and barcode validation using various OTU picking criteria, viz., BM, BCM, and ASB. Species discrimination was performed with a 3.0% threshold, where 20 (1.79%) sequences in rbcL and 40 (14.44%) in matK were without any match close to the threshold. Those sequences violating the threshold value exhibited incorrect or ambiguous matches in seven species (accessions, *n* = 19) and 19 species (*n* = 40) belonging to rbcL and matK datasets. However, in rbcL, there were 934 (83.54%) sequences, and in matK, there were 177 (63.89%) sequences with the closest match of 0% threshold. According to the criteria considered for sequence match, BM and BCM altogether revealed the taxonomic resolution of 54.83% and 54.74% for rbcL and 61.37% for matK within the threshold (Table 1). For ASB, rbcL and matK exhibited an accuracy of 58.68% and 52.35%, respectively (Table 1).

Identification at the species level exhibited correct species match for 170 (41.06%) species in the rbcL dataset, of which only 118 (28.50%) were recognized as true species. Similarly, in the matK dataset 17 (15.04%) species were identified as correct species, while only eight (7.08%) species were recognized as true species (Table 1).

Further, a barcode gap analysis was performed on the GBMA dataset through the ABGD web portal using Jukes-Cantor (JC69), Kimura (K80), and simple distance metrics. The JC69 and K80 model methods showed the highest accuracy for the rbcL dataset by detecting 9 and 4 partitions, respectively, and both the metrics were able to resolve 484 (43.29%) sequences belonging to 122 (29.47%) species (Table 2). For the matK dataset, JC69, K80, and simple distance metrics were able to detect ten partitions; with these metrics, 141 (50.90%) sequences belonging to four (3.54%) species were resolved successfully (Table 2). However, in the rbcL and matK datasets, there were 150 and 88 singleton species, respectively. Still, in the ABGD analysis, they were grouped with other species resulting in incorrect or ambiguous classification (Table 2).

**Figure 2:**
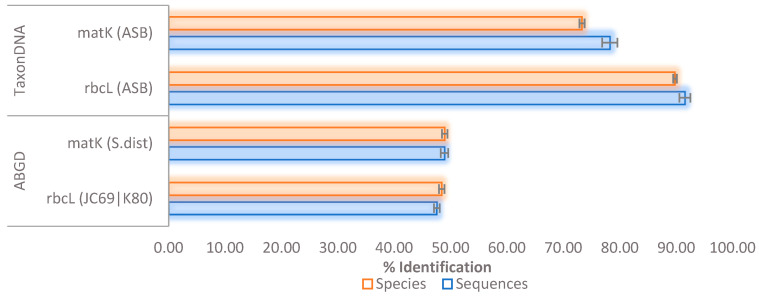
Discrimination potential of ABGD and TaxonDNA’s species identifier using ASB metric for the rbcL and matK barcode markers belonging to FINBOL’s AL-dataset. Abbreviations: ASB: All Species Barcodes, S.dist: Simple Distance.

When the FINBOL dataset was subjected to the barcode validation, the BM and BCM criteria showed the highest accuracy for the matK barcodes (BM = 72.20%; BCM = 72.27%) followed by rbcL (BM and BCM = 55.78%)). However, the ASB criterion revealed contrasting results by showing higher accuracy for the rbcL barcodes (sequence = 91.54%; species = 89.79%) than the matK barcodes (sequence = 78.22%; species = 73.30%) (Figure 2). Moreover, these results were even higher than the ABGD at the sequence and the species level for both rbcL and matK barcode markers, respectively (Figure 2). Furthermore, the TaxonDNA’s ASB metric and ABGD analysis revealed that the difference between the rate of true species recognition and the rate of specimen discrimination was not more than 5%, and this could be attributed to the availability of enough species memberships in the FINBOL dataset (≥3 individuals per species). Whereas in the GBMA dataset, the difference between the rate of true species recognition and specimen discrimination was up to 24%, as observed in the TaxonDNA’s ASB metric for the rbcL barcode marker (Table 1). It could be due to the low specimens per species in the dataset (<2 individuals per species).

### 2.3. Supervised Species Identification and Barcode Validation 

#### 2.3.1. Evaluation of Classifier(s)

**Figure 3:**
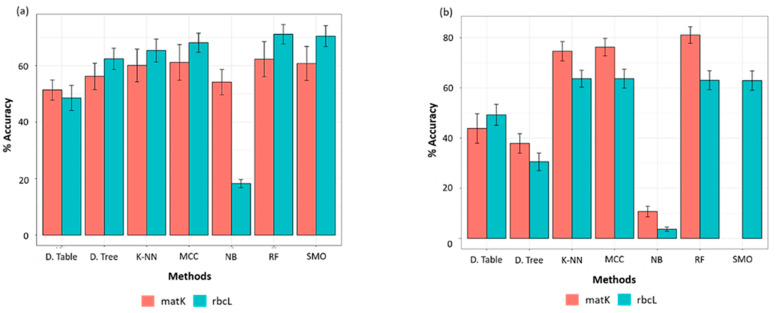
Evaluation of classifiers for their efficiency to discriminate plant barcodes. (**a**) GBMA’s AL dataset; (**b**) FINBOL’s AL dataset. Abbreviations: D. Table: Decision Table (Rules), D. Tree: Decision Tree (Trees), K-NN: K-Nearest Neighbor (Lazy), MCC: Multi-Class Classifier (Meta), N. Bayes: Naïve Bayes (Bayes), RF: Random Forest (Trees), SMO: Sequential Minimal Optimization (Functions).

The machine learning classifiers were evaluated for their performance before their employment using Paired T-Tester (corrected) in the WEKA experimenter. For the GBMA’s AL dataset, in rbcL, the RF classifier estimated the highest accuracy (71.09%, SD = 3.44), followed by SMO classifier (70.43%, SD = 3.69), MCC (68.11%, SD = 3.39) and K-NN (65.33%) (Figure 3). In contrast, other classifiers scored lower and were thus considered unfit for further analysis (Table 3). In matK, the RF classifier predicted the highest accuracy (62.28%, SD = 6.21), followed by MCC (61.12%, SD = 6.30), SMO (60.76%, SD = 6.03), and K-NN (60.04%, SD = 5.8) (Figure 3a).

However, contrasting results were observed for FINBOL’s AL dataset in terms of accuracy of barcode markers in species discrimination. The matK scored the highest accuracy compared to that of rbcL (Figure 3b), though the performance among the classifiers was somewhat similar, as observed in the GBMA dataset for the matK marker. It was seen that the RF classifier (81.05%, SD = 3.33) in matK exhibited the highest accuracy, followed by MCC (76.25%, SD = 3.51), and then K-NN (74.59%, SD = 3.85). Unfortunately, we were not able to plot the performance of SMO due to computational restrictions, as it could not be completed on the server (40 cores, 16 GB RAM per core) even after running for ten days. For the rbcL dataset, MCC and K-NN (63.67%, SD = 3.77) scored the highest accuracy, followed by RF (63.03%, SD = 3.78) and SMO (62.90%, SD = 3.83).

Furthermore, the GBMA’s alignment-free dataset was evaluated at different k values (k-mer lengths: k = 2, k = 3, k = 4, k = 5, and k = 6) and nearest neighbors (1-NN, 3-NN, and 5-NN) for rbcL and matK barcode markers (Table 3). It was observed that the rbcL dataset exhibited the highest species discrimination of 71.55% (RMSE = 0.0339) at 1-NN and k = 5 using the gappy kernel, followed by LAF at 68.15% (RMSE = 0.0353) at k = 5 (Table 3). For the matK dataset, the highest accuracy was observed at 1-NN for the gappy kernel with 61.73% (RMSE = 0.0736) at k = 3, followed by mismatch kernel with 61.01% (RMSE = 0.0725) at k = 6 (Table 3). Accordingly, SML classifiers were employed for only those rbcL and matK AF datasets exhibiting the highest resolution potential with respective k and K-NN sizes. For FINBOL’s alignment-free dataset, an intermediate k-mer size of four was used for employing the classifiers, and thus evaluation of classifiers on FINBOL’s alignment-free dataset was not performed.

#### 2.3.2. Employing Classifiers for Analysis

The SML analysis using the Random Forest classifier exhibited the highest rate of accurate identification of 71.11% (RMSE = 0.0316) for rbcL sequences belonging to 54.35% species, and 62.45% (RMSE = 0.0643) of sequences of matK belonging to 15.93% species (Table 4). The alignment-free analysis using SML classifiers exhibited the highest species resolution for gappy kernel using the K-NN classifier for rbcL to resolve 71.55% (RMSE = 0.0339) sequences at k = 5 belonging to 57.25% species, and matK for 61.73% (RMSE = 0.0736) sequences at k = 3, belonging to 15.93% species (Table 4). Overall, the species with more than two individuals (*n*) exhibited the highest rate of correct identifications compared to those species with *n* = 1.

**Figure 4:**
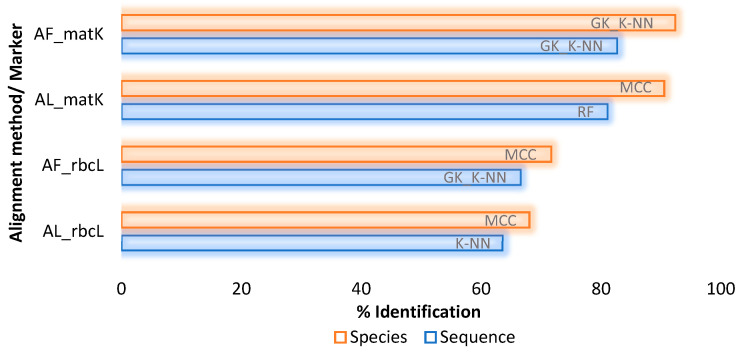
Best performing SML classifiers to resolve sequences and species from the rbcL and matK markers belonging to the FINBOL’s AL and AF dataset. Classifiers employed: MCC: Multi-Class Classifier, RF: Random Forest, K-NN: K-Nearest Neighbor, GK_K-NN: gappy kernel with K-Nearest Neighbor.

The analysis of FINBOL’s AL dataset using SML algorithms revealed the highest accuracy of 81.07% (RMSE = 0.0265) for the matK barcodes using RF classifier (Figure 4); however, for the GBMA dataset, the highest accuracy was observed for rbcL barcodes (Table 4). At the species level, the MCC classifier achieved the highest accuracy for the FINBOL dataset at 90.58% for the matK barcodes (Figure 4). In the case of alignment-free analysis, the FINBOL dataset exhibited the highest rate of species resolution of 82.66% (RMSE = 0.0278) belonging to 92.41% species for gappy kernel using the K-NN classifier (Figure 4). Again, the highest rate of barcode discrimination and species resolution for the FINBOL compared to the GBMA can be correlated to the species to specimen ratio, as the FINBOL dataset possesses enough species memberships with ≥3 individuals per species.

#### 2.3.3. Accuracy of Machine Learning Classifiers

**Figure 5:**
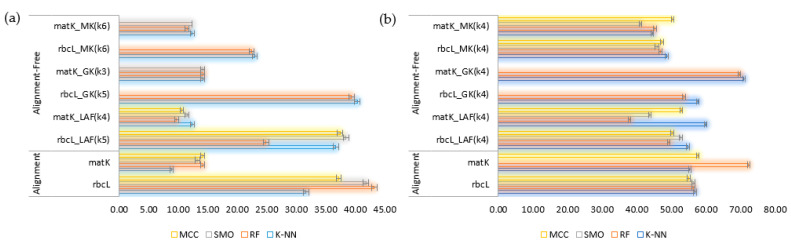
Accuracy of classifiers to determine true positive species from (**a**) GBMA, (**b**) FINBOL datasets based on alignment and alignment-free methods. Classifiers employed: MCC: Multi-Class Classifier, SMO: Sequential Minimal Optimization, RF: Random Forest, K-NN: K-Nearest Neighbor.

The True Positive Rate (TPR) and False Positive Rate (FPR) revealed detailed accuracy of classifiers employed for species identification. For the GBMA’s rbcL barcodes, a Random Forest classifier exhibited the highest true positive rate (TPR = 1.0) for 43.24% (SD = 0.476) of species in an aligned set of sequences. In contrast, for the alignment-free sequence set, K-NN was able to resolve 40.34% (SD = 0.49) of species accurately at TPR = 1.0 (Figure 5a) through the gappy kernel approach. For matK, Random Forest and Multi-Class classifiers showed the highest true positive rate (TPR = 1.0) for 14.16% (SD = 0.35) species in the AL dataset and using a gappy kernel approach in AF datasets, respectively (Figure 5a).

For the FINBOL’s AL dataset, a Random Forest classifier showed the highest true positive rate (TPR = 1) of 72.25% (SD = 0.45) for the matK barcode marker, while for the rbcL marker, the K-NN classifier exhibited the highest TPR of 56.81% (SD = 0.50) (Figure 5b). In the AF dataset, a K-NN classifier using gappy kernel showed the highest TPR of 70.94% (SD = 0.45) for the matK barcode marker, while for the rbcL marker, the K-NN classifier exhibited the highest TPR of 57.59% (SD = 0.49) (Figure 5b).

Overall, considering the performance of unsupervised and supervised learning methods on both datasets, these results confirm that the supervised classifiers K-NN and RF are robust enough to show the highest rate of species identification and true species recognition. Following the two classifiers, the multi-class classifier is the one to show high species resolution, though its performance declined when employed to recognize true species (Figure 4 and Figure 5). However, outstanding results were obtained using unsupervised methods on the standardized dataset, as the TaxonDNA’s ASB criterion showed enough species resolution and even true species recognition potential for the rbcL marker. In the case of barcode markers, from the results obtained using the FINBOL dataset, the matK marker showed higher resolution potential than the rbcL marker. However, this was not the case for GBMA’s matK marker dataset when analyzed through the SML method, as it lacks enough known species memberships compared to the rbcL dataset. Apart from the barcode markers, the difference between the accuracy of alignment-based and alignment-free (gappy kernel) techniques was not more than 2–4% in both datasets.

## 3. Discussion

Species identification through ‘DNA barcoding’ relies upon the principle that interspecific divergence sufficiently outscores intraspecific divergence, with a threshold value demarcating the biological species [54]. Usually, a 3% threshold is sufficient to distinguish congeneric species [54]; however, this threshold can also be calculated from genetic distances instead of using a single arbitrary 1% or 3% [37,55,56]. In our study, for the GBMA dataset we used a 3% threshold for species delimitation for rbcL and matK sequences, where the ASB and BM criteria of TaxonDNA performed better than BCM (Table 1). Some major studies from the Arabian Peninsula demonstrated species resolution ranging from 58% for rbcL to 35% for matK marker: from UAE, Maloukh et al. [31] reported about 100% resolution for rbcL and 35% for matK in 51 species and Abdullah [9] from Kuwait, reported 58% and 69% for 244 species using rbcL and ITS2 markers, respectively. The CBOL working group also demonstrated that the species discrimination potential for rbcL and matK barcode marker is not more than 70% [16].

Similarly, for the FINBOL dataset, the ASB criterion effectively resolved species belonging to both the barcode markers. However, contrasting results were observed between the ASB and BM and BCM metrics, where BM and BCM showed higher accuracy for the matK barcodes, whereas the ASB metric exhibited the highest accuracy for rbcL (Figure 2). We first believed this could be due to the lack of enough species memberships in matK, as observed for the GBMA dataset (Table 1). However, after analyzing the FINBOL dataset with enough sequences per species, this could be due to the gaps, missing data, and/or uneven sequence length in the matK dataset, thus causing the ASB metric to recognize a comparatively higher number of species as ambiguous or incorrect. Many studies have demonstrated that the missing data [57,58] or variable sequence length [59,60] might affect the rate of species estimation. 

Recent studies have preferred to use the difference between minimum interspecific and maximum intraspecific divergence to define the barcode gap [40,61]. This was found to be more efficient over the use of mean intra- and inter-specific divergence [38]. Our analysis on the GBMA (rbcL and matK) and FINBOL (rbcL) datasets showed that the ABGD’s JC69 metric was much more effective in species discrimination, though the simple distance metric was observed to be effective only for the FINBOL’s matK dataset. At the GBMA’s species-level analysis, species overlap was seen in 77 species of rbcL and six species of matK as they merged. Moreover, one or more groups of species was recognized in 117 species of rbcL and 76 species of matK, further demonstrating incorrect or ambiguous identification, respectively. Usually, the absence of the barcode gap leads to the merging of different species, whereas high genetic divergence leads to the splitting of a species group [40].

While genetic distance-based OTU picking methods, which use intraspecific thresholds and barcode gaps, are efficient, character-based methods have also been shown to be competent over time for accurate species identification [41,62]. Our study used alignment and alignment-free methods to analyze sequences using character-based machine learning algorithms.

Compared to the unsupervised methods, the alignment and alignment-free SML analysis on the GBMA dataset exhibited higher accuracy to discriminate barcode sequences, as well as species (Table 4, Figure 5a). Accordingly, the AL-based RF classifier showed the highest accuracy to determine true positive species, followed by the AF-based K-NN classifier through the gappy kernel approach (Figure 5a). However, for the FINBOL dataset, the unsupervised method TaxonDNA’s ASB criterion exhibited higher accuracy than SML to determine true positive species. This could be understood from the technique (95th percentile) behind the ASB criterion. It tends to identify queries more rigorously, where conspecifics within the 95th percentile of all intraspecific distances are considered successful identifications. Here the ASB criterion utilizes information from all conspecifics in the database, and therefore, if we have enough known sequences from a single species, the identifier will be more confident in assigning this species’ name to the query. However, a very biased sample of conspecific or congeneric sequences could affect the rate of accurate identification [37].

**Figure 6:**
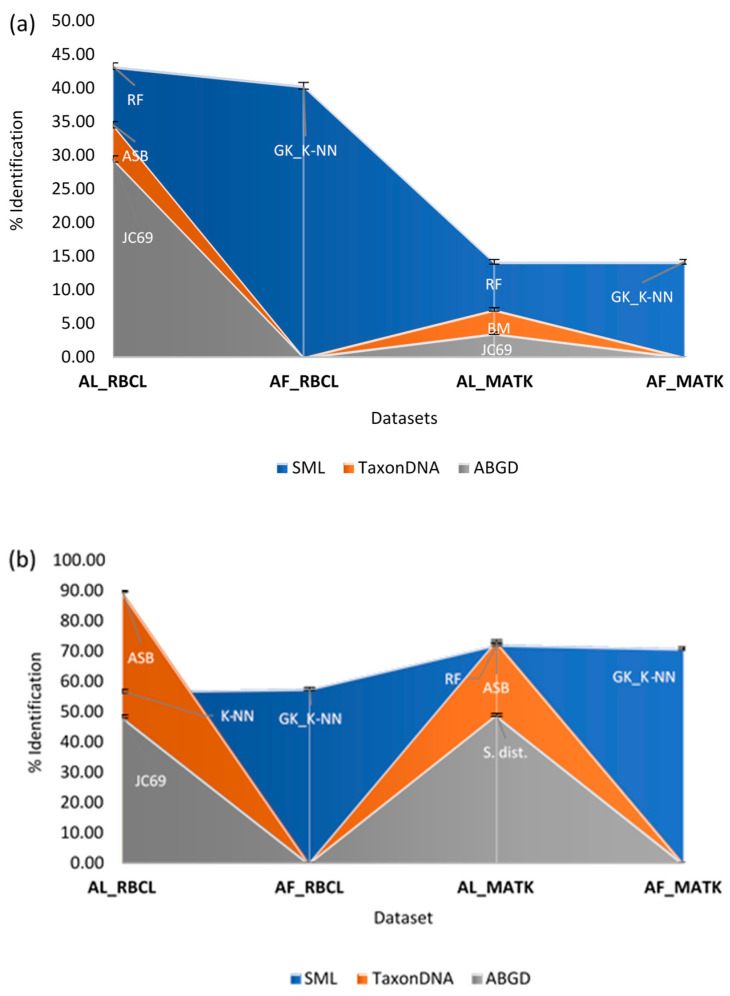
Discrimination potential of various methods employed for AL and AF datasets of rbcL and matK markers. (**a**) GBMA dataset, (**b**) FINBOL dataset. Captions: GK_K-NN: gappy kernel with K-Nearest Neighbor Classifier; MCC: Multi-Class Classifier, RF: Random Forest, ASB: All Species Barcode Metric, S. dist: Simple Distance Metric, JC69: Jukes-Cantor Metric.

Indeed, the ASB is a conservative identifier that would probably assign a species name if the query was followed by all known barcodes, so it is required to have at least two conspecific matches [37]. Thus, for any two or more datasets, the dataset (e.g., FINBOL) having a greater sample size per species will show higher species resolution than the other sets (e.g., GBMA). Nevertheless, the TaxonDNA’s ASB criterion performs better in true species recognition than the SML methods. Apparently, SML lacks the ASB’s 95th percentile approach (Figure 6b), demonstrating the robustness of the ASB criterion towards accurate species identification. Tan et al. [63] observed that the distance-based ASB criterion is much stricter than its neighboring criteria, the BM and BCM, thus reflecting taxonomic comprehension of relatively known taxa in a much better way. Accordingly, the highest identification rate can be seen for the ASB compared to the BM and BCM criteria, which was observed for the rbcL barcodes followed by matK (FINBOL dataset). Similar studies employing various barcode markers have observed a higher rate of species discrimination for the ASB compared to the BM and BCM metrics [64,65,66,67,68]. Considering the efficiency of barcode markers, the BM and BCM metrics and the ABGD and SML (AL and AF) methods showed higher efficiency in resolving matK barcodes. Those methods successfully resolved species and recognized true species from matK barcodes compared to the rbcL, as suggested by ASB (Figure 3, Figure 4 and Figure 6b). Overall, the TaxonDNA’s ASB criterion can efficiently discriminate well-aligned barcodes like rbcL compared to the barcodes with gaps in matK, further predicting the highest correct identifications for rbcL and comparatively lowering the number of correct identifications for the matK (Figure 4 and Figure 6a,b).

Moreover, the skewed rate of identification in matK could be primarily due to the gaps or missing data; secondly, it could also be due to the higher evolutionary rate, which is about 2–3 times higher than rbcL [69], giving high discriminatory power and sufficient reliability. Similar studies on plant barcodes have validated the efficacy of the matK marker for species discrimination [70,71,72].

Although the GBMA dataset has a low specimen count per species, the SML methods showed better performance than the unsupervised methods. Overall, for determining true species, the RF (for AL) and K-NN classifiers (for the AF/AL) are far more robust than other supervised and unsupervised methods (Figure 5a). Thus, we recommend evaluating the performance of the classifiers before implementing them through the AL- or AF-based approach. For the AF-based approach, K-NN-based evaluation can be implemented. The selection of suitable parameters is essential for the K-NN along with the appropriate k-mer size. This was demonstrated on the GBMA dataset, where the range of Nearest Neighbor (NN) values from 1 to 3 was tested on series of k-mer lengths (k) from 2 to 6, where 1-NN showed the highest accuracy at k = 5 for rbcL and k = 3 for matK. Similarly, Kuksa et al. [73] observed that the error rates in the nearest neighbor classifier increased with the increase in the nearest neighbor values.

**Figure 7:**
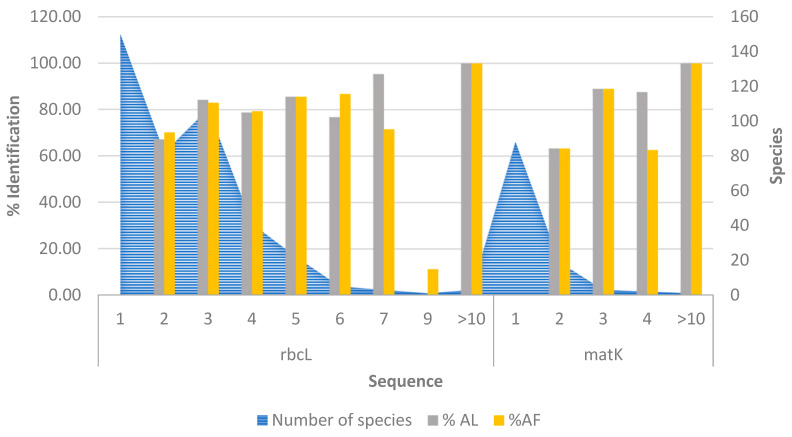
Species identification success rate for the GBMA dataset based on the number of sequences per species. Abbreviations: AL: Alignment-Based, AF: Alignment-Free.

In congruence to our observations, Weitschek et al. [41,43] observed high performance for the SMO classifier using the aligned dataset and K-NN classifier for the alignment-free dataset; however, they did not evaluate the RF classifier. Similar studies have demonstrated the efficient performance of SML algorithms in species resolution [41,43,74,75,76]. Moreover, if >1 attribute per species is provided, then the accuracy of these classifiers significantly increases to achieve still higher rates for species discrimination. However, the success rates may be skewed for the datasets like GBMA with low specimen count per species, where the rbcL and matK sets have about 36.23% and 77.87% singleton species, respectively. The success rate may increase with the number of sequences per species, which was observed in the GBMA’s rbcL set, with an exception for one species (*Tetraena propinqua*) having nine individuals, as this species tends to exhibit incorrect identification for all classifiers (Figure 7). Thus, it has been suggested that, the species must have a prior known membership for the SML based identification to allow correct identification [41,76].

Overall, the comparative assessment of unsupervised and supervised techniques suggested that the TaxonDNA’s ASB criterion is much more efficient to resolve aligned datasets with high TPR when there are enough known specimens per species. Otherwise, for the unaligned datasets with gaps and missing data, the SML method with RF and GK_K-NN classifiers followed by MCC are well-suited classifiers to obtain high resolution at the sequence and the species level (Table 4, Figure 4). Moreover, RF or GK_K-NN classifiers are efficient enough to detect true positive species, as demonstrated (Table 4, Figure 6a,b). At the marker level, our analysis suggests that matK tends to exhibit higher identification rates compared to the rbcL, though the GBMA dataset has shown contrasting results due to low species memberships. The number of singleton species is higher in matK, which may have affected the rate of species estimation (Figure 6).

## 4. Materials and Methods

**Figure 8:**
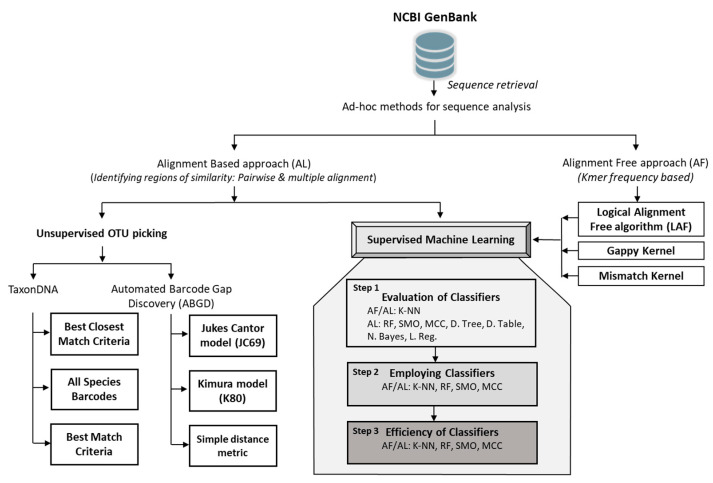
The workflow of the comparative approach implemented for analyzing plant DNA barcodes of Arabia. Abbreviations: D. Table: Decision Table (Rules), D. Tree: Decision Tree (Trees), K-NN: K-Nearest Neighbor (Lazy), MCC: Multi-Class Classifier (Meta), N. Bayes: Naïve Bayes (Bayes), RF: Random Forest (Trees), SMO: Sequential Minimal Optimization (Functions).

Plant DNA sequences from the Arabian Peninsula, published between 2009–2019, belonging to two barcode regions rbcL and matK, were extracted from NCBI GenBank. The initial regulatory criteria for sequence retrieval were implemented, where more than 400 bp sequences were considered standard barcodes [13]. Thus, the experimental dataset (GBMA) with a total of 1502 sequences was prepared and preprocessed to generate two forms of datasets, ‘Alignment’ (AL) and ‘Alignment-Free’ (AF) (Figure 8). The standardized dataset (FINBOL) was prepared from a total of 4810 plant barcodes (rbcL and matK) that were retrieved from the BOLD System’s public project DS-FBPL1 (https://doi.org/10.5883/ds-fbpl1, accessed on 22 September 2021). The sequences obtained were then sorted using Python 3 for equivalent distribution of individuals and species by considering a criterion of at least three individuals per species for both the barcode markers (rbcL and matK). The FINBOL dataset was further preprocessed to generate two forms of datasets, ‘Alignment’ (AL) and ‘Alignment-Free’ (AF) (Figure 8).

### 4.1. Pre-Processing

The alignment datasets were prepared by achieving the best possible alignment through Geneious Prime v11.0.3. Those sequences that failed to align were eliminated, the alignment was trimmed, and the maximum sequence length of 582 base pairs (bp) was obtained for the rbcL and 1112 bp for matK. Further re-alignment was done to achieve highest possible pairwise identity (GBMA: rbcL = 90% and matK = 80.6%; FINBOL: rbcL = 90.4% and matK = 74.5%). The alignment-free datasets were prepared directly from the retrieved sequences and those representing the AL datasets. 

The final experimental dataset (GBMA) consisted of 1118 (rbcL) and 227 (matK) sequences (see Additional File 1), while the final standardized dataset (FINBOL) had a total of 1194 sequences belonging to each of the rbcL and matK barcode markers, respectively. As the efficiency of the analytical method exclusively relies upon the alignment, both alignment and alignment-free methods were adopted for our analysis (Figure 8).

For the alignment-based method, sequences were converted from aligned character strings into numeric vector form through FASTA to WEKA converter [41]. In the case of alignment-free methods, K-mer frequencies from K-mer size k = 2 to k = 6 were generated using the Python (v3.7.4)-based Logical Alignment Free (LAF) algorithm [43] in Linux (Ubuntu 18.0) and Python-based string kernel methods. The python scripts are available at GitHub (https://github.com/jakob-he/string-kernel, accessed on 22 September 2021), viz., gappy kernel and mismatch kernel on Windows 10 [42,48]. For ‘GappyKernel’, the following parameters were provided as inputs in the string kernel method: (1) class ‘k’, representing the length of k-mers, which was tested from 2 to 6 in this study, (2) ‘g’, which represents the integer gaps allowed in k-mers; for ‘g’ parameters, we tested values from 0 to the highest number of gaps in our dataset. However, the value for the lowest number of gaps (g = 1) exhibited the highest correct classifications by consuming a significantly low amount of memory and thus was used for analysis. (3) ‘t’, which represents the alphabet/value indicating the sequence type. For the DNA type, the value of ‘t’ was set to 0. In the ‘MismatchKernel’ class, the following parameters were set: (1) ‘l’ was set to 4 as the length of the alphabet for DNA sequence, (2) while ‘k’ was the length of k-mers, which was tested from 2 to 6, and (3) ‘m’ represents mismatch in themers of sequences that were considered as m = 1; normally small values of m should work well. The complexity of the algorithm is exponential in m. In order to facilitate ease of use of string kernel methods for DNA barcode datasets, we created python scripts for generating k-mers using string and gappy kernel, which could be executed in windows command-line tool (https://github.com/BioInf2305/ML-Barcoding, accessed on 22 September 2021). Further, the obtained aligned datasets with numeric vector strings and alignment-free datasets with k-mer frequencies were analyzed by various classifiers with ten-folds of cross validation. The analysis was performed using Waikato Environment for Knowledge Analysis (WEKA), a suite of machine learning software written in ‘JavaWEKA’ [47,53].

### 4.2. Sequence Analysis

The aligned sets (rbcL-AL and matK-AL) were used for species identification and validation of barcodes using an unsupervised OTU picking method based on pairwise genetic distance, further exploring their intra- and inter-specific genetic divergence through TaxonDNA v1.9 Species Identifier [37]. In TaxonDNA, species discrimination was done using three different criteria, ‘Best Match’ (BM), ‘Best Closest Match’ (BCM), and ‘All Species Barcodes’ (ASB) [37]. Those three criteria are designed to work under different circumstances. The BM is the least stringent criterion, where the query is assigned with the species name based on its best matching barcode, regardless of the similarity to the barcode sequence. This issue has been avoided in the BCM criterion, where conspecifics within the 95th percentile of all intraspecific distances are considered successful identifications. The last criterion (ASB) identifies queries more rigorously and is an application of the BCM strategy. Here the criterion utilizes information from all conspecifics in the database, which assists the identifier in assigning the species name to the query with more confidence. The categorization of species and sequences was done based on comments provided by the respective criterion. Those sequences recognized as correct by BM/BCM/ASB criteria were tagged as CORRECT regardless of its species member in the ‘Correct’/’Incorrect’/’Ambiguous’ category. Those species representing their sequences under the Correct category, with no sequence in any other category (Incorrect/Ambiguous), were tagged as TRUE SPECIES. The barcode gap analysis was performed for both the datasets using the ABGD web server (wwwabi.snv.jussieu.fr) [40], where the Jukes-Cantor model (JC69), Kimura (K80) (TS/TV = 2.0), and simple distance metrics were executed with the following settings: Pmin = 0.001, Pmax = 0.1, relative gap width (X) = 1.5, and Nb bins = 20. Further, recognition of the initial partition was done at the relevant prior maximal distance based on the group having the most relevant number of OTUs. Then the groups obtained in the initial partition were sorted depending upon the following criteria: If the same species were grouped together and not found in any other group, then they were tagged as TRUE; if the same species were grouped together but also found in another group they were tagged as AMBIGUOUS; if multiple species were grouped together then they were tagged as INCORRECT; if only one individual of a species represented a group, then it was tagged as a SINGLETON.

Further, machine learning algorithms for species identification were implemented in WEKA v3.8.2 using three steps. At the first step, selection of classifiers was done from almost every approach viz., functions (SMO) [51,52], trees (Random Forest (RF) [50], and decision tree (J48) [60]), Bayes ((Naïve Bayes)) [77], lazy (K-Nearest Neighbor (K-NN) [49]), rules (decision table) [78], meta (classification via regression and Multi-Class Classifier (MCC) [53]). Moreover, these classifiers were evaluated in the WEKA experimental environment [53], with ten-fold CV and ten iterations (Figure 6, Table S1). Then they were tested using Paired T-Tester (Corrected) with two-tailed confidence (C = 0.05) for a percentage of correct classification for each classifier. Only four classifiers with the highest accuracy were selected and employed in the second step for analysis (Figure 6). In the case of GBMA alignment-free datasets, K-mer frequencies generated using LAF and string kernels were initially evaluated for their performance in identifying Nearest Neighbor (NN) at different intervals (NN = 1, 3, and 5) using the K-NN classifier. However, the string kernel requires high computation power; thus, we could not implement it for some classifiers. Further, based on the performance of the K-NN classifier, those alignment-free datasets exhibiting the highest correct classifications were chosen for the SML analysis. In FINBOL alignment-free datasets, K-mer frequencies and nearest neighbor intervals were kept default (k = 4 and NN= 1). The selected SML classifiers were then employed 10-folds with the CV for AL and AF datasets. The parameters for the SML classifiers were kept almost at default, including the batch size of 100, which is the percent of the training set size. Moreover, the K-NN classifier was employed for both AL and AF datasets. The GBMA’s AF dataset was tested at various NN intervals (1, 3, and 5) (weka.classifiers.lazy.IBk -K 1/3/5 -W 0), while the AL dataset was analyzed only with 1-NN. Additional settings included implementation of nearest neighbor search algorithm with the ‘linear nearest neighbor search’ (-A ‘weka.core.neighboursearch.LinearNNSearch’) using ‘Euclidean distance’ similarity function (-A\’weka.core.EuclideanDistance -R first-last\’). The RF classifier was implemented with the default bag size percent of 100 (percentage of training set size), also ‘the number of iterations’ was kept to a default of 100 (RandomForest -p 100 -I 100 -num-slots 1 -K 0 -M 1.0 -V 0.001 -S 1). For SVM’s SMO classifier, the filter type used was ‘normalize training data’ and the ‘numfolds’ parameter was kept at ‘-1’. The number of folds for CV was used to generate training data for calibration of models (weka.classifiers.functions.SMO -C 1.0 -l 0.001 -p 1.0E-12 -N 0 -V -1 -W 1 -K). Along with this, polynomial kernel was chosen (weka.classifiers.functions.supportVector.PolyKernel -E 1.0 -C 250007) and the calibrator class used was multinomial logistic regression model with a ridge estimator (weka.classifiers.functions.Logistic -R 1.0E-8 -M -1 -num-decimal-places 4). The multi-class classifier was implemented (weka.classifiers.meta.MultiClassClassifier -M 0 -R 2.0 -S 1) by choosing the multinomial logistic regression model as the base classifier with a ridge estimator (-W weka.classifiers.functions.Logistic -R 1.0E-8 -M -1 -num-decimal-places 4). Moreover, to know the classifier’s performance, ‘RMSE’ (Root Mean Squared Error) is generally used as it represents the sample standard deviation of the differences between predicted values and observed values.

## 5. Conclusions

We employed different ad-hoc methods to assess Arabian plant barcodes, where two widely implemented unsupervised OTU picking and supervised learning methods were demonstrated. However, the Arabian plant barcode dataset (GBMA) lacks enough conspecifics. Thus, a standard curated dataset (FINBOL) was obtained from BOLD Systems and analyzed side-by-side to understand the performance of methods and markers employed. Our analysis suggests that the well-aligned datasets with enough conspecifics (≥3 specimens per species), as in FINBOL, could help achieve the highest rate of accurate species identification, as observed using the TaxonDNA’s ASB criterion. The alignment-free datasets with gaps or missing data like matK or those with a low species to specimen ratio, as in the GBMA dataset (for rbcL and matK), could perform well when analyzed through SML methods. Moreover, the gappy kernel approach assisted by the K-NN classifier could be employed for resolving barcodes from the AF datasets, followed by the RF classifier which performed well for both markers belonging to the AL datasets. Nonetheless, the MCC classifier showed high species resolution, though its performance declined when employed to recognize the true species.

Altogether, our Arabian plant barcode datasets (rbcL and matK) primarily lack sufficient species membership for both the markers, leading to skewed species identification. However, the FINBOL dataset, when analyzed, revealed the necessity of conspecifics to obtain the high-resolution potential, further highlighting the requirement to generate enough DNA barcodes to build a robust DNA barcode library for Arabian plants. With this concern, we have already engaged ourselves in barcoding the vascular plants from the United Arab Emirates through the projects BAEF (vascular plants) and BEMP (medicinal plants) on the BOLD systems. The barcode data and other related data from our projects will be made public soon afterwards. We would encourage taxonomists from Arabian countries to join our Arabian Barcode of Life (ARABOL) campaign at the BOLD systems and contribute to the Arabian Plant Barcode resource. We hope this campaign will generate more plant barcodes, including other important barcodes from ITS2 and psbA-trnH that have not been covered in this study. Moreover, enough barcode data will help us demonstrate other essential barcode markers (ITS2 and psbA-trnH) in the near future.

Furthermore, the curated Arabian plant barcodes in this study could offer assistance in the form of a reference library to improve the DNA barcode identification success rate for the Arabian plants. The SML workflow provided could also assist plant taxonomists in selecting the efficient approach to achieve better species resolution.

## Figures and Tables

**Table 1:** Discrimination potential of TaxonDNA’s species identifier for the rbcL and matK barcode markers belonging to GBMA’s AL-dataset.

Identification		rbcL			matK		
		BM	BCM	ASB	BM	BCM	ASB
Sequences (rbcL = 1118; matK = 277)	Correct	54.83	54.74	58.68	61.37	61.37	52.35
	Incorrect	14.31	12.79	6.80	31.04	18.05	2.89
	Ambiguous	30.86	30.68	32.74	7.59	6.14	30.32
	No match (@3% Threshold)	*NA*	1.79	1.79	*NA*	14.44	14.44
Species (rbcL = 414; matK = 113)	Correct	41.06	NA	35.99	15.04	NA	4.42
	True species	28.50	NA	34.54	7.08	NA	3.54

Abbreviations: BM: Best Match, BCM: Best Closest Match, ASB: All Species Barcodes and NA: Not Available.

**Table 2:** Discrimination potential of ABGD distance metrics for the rbcL and matK barcode markers belonging to GBMA’s AL-dataset.

Barcode Marker	Distance Method	Partitions	Partition at Prior Maximal Distance (P)	Groups	Correct	Incorrect	Ambiguous	Singleton	True Species
rbcL (species = 414; sequences = 1118)	Jukes-Cantor (JC69)	9	0.002783	411	43.29	25.04	22.90	8.77	29.47
	Kimura (K80)	4	0.002783	411	43.29	25.04	22.90	8.77	29.47
	Simple distance	9	0.002783	248	31.93	53.22	9.12	5.72	18.36
matK (Species = 113; sequences = 277)	Jukes-Cantor (JC69)	10	0.0359	52	50.90	34.66	4.69	9.75	3.54
	Kimura (K80)	10	0.0359	52	50.90	34.66	4.69	9.75	3.54
	Simple distance	10	0.0359	52	50.90	34.66	4.69	9.75	3.54

**Table 3:** Classification performance of the alignment-free methods for the GBMA’s AF dataset are shown in the form of a heat map table at various k-mer lengths using the nearest neighbor approach.

Method	k = 2		k = 3		k = 4		k = 5		k = 6	
	rbcL	matK	rbcL	matK	rbcL	matK	rbcL	matK	rbcL	matK
**1-NN**										
LAF	49.19	51.98	52.14	57.76	66.99	60.64	68.15	59.92	67.26	58.84
	(0.0436)	(0.0812)	(0.0424)	(0.0767)	(0.0359)	(0.0744)	(0.0353)	(0.0751)	(0.0357)	(0.0759)
Gappy kernel	69.14	61.37	70.84	61.73	71.19	61.37	71.55	61.37	NA	61.37
	(0.0351)	(0.0739)	(0.0343)	(0.0736)	(0.0341)	(0.0738)	(0.0339)	(0.0738)		(0.0737)
Mismatch kernel	47.04	55.59	54.38	59.2	57.6	60.28	58.85	59.56	59.74	61.01
	(0.0445)	(0.0769)	(0.0416)	(0.0737)	(0.0402)	(0.0731)	(0.0397)	(0.0735)	(0.0393)	(0.0725)
**3-NN**										
LAF	34.25	51.26	41.05	53.79	56.08	55.23	59.21	55.59	55.72	54.15
	(0.0446)	(0.0756)	(0.0427)	(0.0725)	(0.0377)	(0.0714)	(0.0372)	(0.072)	(0.0381)	(0.0725)
Gappy kernel	58.76	55.95	61.71	55.23	61.44	54.87	61.53	54.51	NA	54.51
	(0.0373)	(0.0713)	(0.0365)	(0.0714)	(0.0364)	(0.0714)	(0.0364)	(0.0715)		(0.0717)
Mismatch kernel	37.74	51.98	40.51	55.23	43.2	57.4	45.08	55.23	45.52	55.95
	(0.0447)	(0.0739)	(0.0425)	(0.0715)	(0.0418)	(0.0708)	(0.0414)	(0.0714)	(0.0412)	(0.0708)
**5-NN**										
LAF	29.78	50.9	36.4	51.98	46.6	55.95	46.6	54.51	43.55	53.79
	(0.0451)	(0.0742)	(0.0432)	(0.0716)	(0.04)	(0.0701)	(0.0399)	(0.0704)	(0.0405)	(0.0714)
Gappy kernel	47.76	54.15	48.56	55.95	48.39	54.87	48.3	54.15	NA	54.15
	(0.0399)	(0.0704)	(0.0395)	(0.0701)	(0.0395)	(0.0704)	(0.0394)	(0.0706)		(0.0711)
Mismatch kernel	30.41	52.34	34.43	53.79	36.49	55.95	38.46	53.79	38.72	54.87
	(0.045)	(0.0733)	(0.0436)	(0.0707)	(0.0431)	(0.0700)	(0.0428)	(0.0707)	(0.0426)	(0.0699)

Abbreviations: NN: Nearest Neighbor, LAF: Logical Alignment-Free Algorithm, NA: Not Available.

**Table 4:** Performance of SML classifiers for the rbcL and matK barcode markers belonging to GBMA’s AL and AF dataset are shown in the form of a heat map table.

Method		Alignment (%)		Alignment-Free (%)					
				Logical Alignment Free (LAF)		Gappy Kernel (GK)		Mismatch Kernel (MK)	
		rbcL	matK	rbcL (k = 5)	matK (k = 4)	rbcL (k = 5)	matK (k = 3)	rbcL (k = 6)	matK (k = 6)
K-nearest Neighbor (IBK)	Sequences	65.03	59.93	68.15	60.65	71.55	61.73	59.74	61.01
		(0.0357)	(0.0727)	(0.0353)	(0.0744)	(0.0339)	(0.0736)	(0.0393)	(0.0725)
	Species	53.38	15.93	55.80	15.04	57.25	15.93	50.24	15.93
Random Forest	Sequences	71.11	62.45	60.91	59.21	71.19	61.37	59.12	60.64
		(0.0316)	(0.0643)	(0.0366)	(0.0655)	(0.0321)	(0.064)	(0.0364)	(0.0646)
	Species	54.35	15.93	52.17	14.16	58.21	15.04	50.24	15.93
Support Vector Machine (SMO)	Sequences	70.30	61.01	69.76	61.01	NA	61.37	NA	61.01
		(0.049)	(0.0935)	(0.049)	(0.0935)	(NA)	(0.0935)	(NA)	(0.0935)
	Species	54.11	15.04	54.35	15.04	NA	15.04	NA	15.93
Multi-Class Classifier	Sequences	68.34	61.37	69.49	58.84	NA	NA	NA	NA
		(0.0349)	(0.0773)	(0.0359)	(0.0781)				
	Species	54.83	15.04	56.52	13.27	NA	NA	NA	NA

Abbreviations: NA: Not Available.

## Data Availability

Sample accessions retrieved for comparative analysis from NCBI Genbank belonging to rbcL and matK markers are available at the figshare database (https://dx.doi.org/10.6084/m9.figshare.12190965, accessed on 22 September 2021).

## Supplementary Materials

The following are available online at https://www.mdpi.com/article/10.3390/plants10122741/s1, Table S1. Parameters of classifiers that were evaluated for their efficiency to discriminate plant barcodes.
