# Supplementary material for: Evaluation of Arabian Vascular Plant Barcodes (rbcL and matK): Precision of Unsupervised and Supervised Learning Methods towards Accurate Identification
Source: Plants (Basel). 2021 Dec 13;10(12):2741. doi: 10.3390/plants10122741 (PMC8708657; doi:10.3390/plants10122741)
Supplement: Supplementary file 1 [file plants-10-02741-s001.zip › plants-1367435-supplementary.pdf]

Table S1. Parameters of classifiers evaluated for their efficiency to discriminate plant barcodes.

S.no.	Classifier	Parameters tested
1	Functions: SMO	functions.SMO '-C 1.0 -L 0.001 -P 1.0E-12 -N 0 -V -1 -W 1 -K \"functions.supportVector.PolyKernel -E 1.0 -C 250007\" -calibrator \"functions.Logistic -R 1.0E-8 -M -1 -num-decimal-places 4\"' -6585883636378691736
2	Trees: Random Forest	trees.RandomForest '-P 100 -I 100 -num-slots 1 -K 0 -M 1.0 -V 0.001 -S 1' 1116839470751428698
3	Trees: Decision Tree (J48)	bayes.NaiveBayes '' 5995231201785697655
4	Bayes: Naïve Bayes	lazy.IBk '-K 1 -W 0 -A \"weka.core.neighboursearch.LinearNNSearch -A \\\"weka.core.EuclideanDistance -R first-last\\\"\"' -3080186098777067172
5	Lazy: K-NN (IBK)	trees.J48 '-C 0.25 -M 2' -217733168393644444
6	Rules: Decision Table	rules.DecisionTable '-X 1 -S \"BestFirst -D 1 -N 5\"' 2888557078165701326
7	Meta: Classification via Regression	meta.ClassificationViaRegression '-W trees.M5P -- -M 4.0' 4500023123618669859
8	Meta: Multi Class Classifier	meta.MultiClassClassifier '-M 0 -R 2.0 -S 1 -W functions.Logistic -- -R 1.0E-8 -M -1 -num-decimal-places 4' -3879602011542849141
